# Congenital portosystemic venous shunt

**DOI:** 10.1007/s00431-017-3058-x

**Published:** 2017-12-14

**Authors:** M. Papamichail, M. Pizanias, N. Heaton

**Affiliations:** 1 Department of Transplantation and Hepato-Pancreato-Biliary Surgery, Lahey Hospital and Medical Center, Burlington, Boston, MA 01805 USA; 2 Department of Liver Transplantation, Hepatobiliary Pancreatic Surgery, King’s Healthcare Partners, King’s College Hospital NHS FT, Institute of Liver Studies, Denmark Hill, London, SE5 9RS UK

**Keywords:** Congenital, Shunt, Portosystemic

## Abstract

Congenital portosystemic venous shunts are rare developmental anomalies resulting in diversion of portal flow to the systemic circulation and have been divided into extra- and intrahepatic shunts. They occur during liver and systemic venous vascular embryogenesis and are associated with other congenital abnormalities. They carry a higher risk of benign and malignant liver tumors and, if left untreated, can result in significant medical complications including systemic encephalopathy and pulmonary hypertension.

*Conclusion*: This article reviews the various types of congenital portosystemic shunts and their anatomy, pathogenesis, symptomatology, and timing and options of treatment.

**Table:** 

**What is Known:** • *The natural history and basic management of this rare congenital anomaly are presented.*
**What is New:** • *This paper is a comprehensive review; highlights important topics in pathogenesis, clinical symptomatology, and treatment options; and proposes an algorithm in the management of congenital portosystemic shunt disease in order to provide a clear idea to a pediatrician. An effort has been made to emphasize the indications for treatment in the children population and link to the adult group by discussing the consequences of lack of treatment or delayed diagnosis*.

## Introduction

Congenital portosystemic venous shunts (CPSS) are rare vascular anomalies that occur secondary to abnormal development or involution of fetal vasculature. They allow intestinal blood to reach the systemic circulation bypassing the liver, resulting in a variety of symptoms and complications in the longer term [1]. They usually occur as isolated malformations, but multiple shunts may exist. Ascites and portal hypertension are not usually features of CPSS, in contrast to secondary portosystemic shunts in the setting of liver cirrhosis or portal vein occlusion [2]. CPSS are divided into intra- and extrahepatic shunts. Although their clinical manifestations may be similar, the pathophysiology and treatment of the two types differ.

The overall incidence of CPSS is estimated to be 1:30,000 births and 1:50,000 for those that persist beyond early life [3]. The prevalence of intrahepatic shunts is estimated to be 0.0235% as reported from a random ultrasonography screening population sample of asymptomatic adults [4]. In a study of 145,000 newborns in Switzerland, 5 cases of CPSS were identified [5].

Abernethy described a case of an extrahepatic shunt in the postmortem of a 10-month-old female patient in 1793. The portal vein was noted to terminate in the inferior vena cava (IVC) at the level of renal veins. Several other abnormalities were also found in association with the shunt [6].

## Embryology

The primitive liver emerges within an epithelial and mesenchymal interactive network of three basic embryological venous systems (cardinal, vitelline, and umbilical veins). Anterior and posterior cardinal veins and the two vitelline veins comprise the origin of the future systemic and portal venous systems, respectively, whereas the umbilical veins drain the yolk sac and placenta before their final regression [3, 7].

**Fig. 1:**
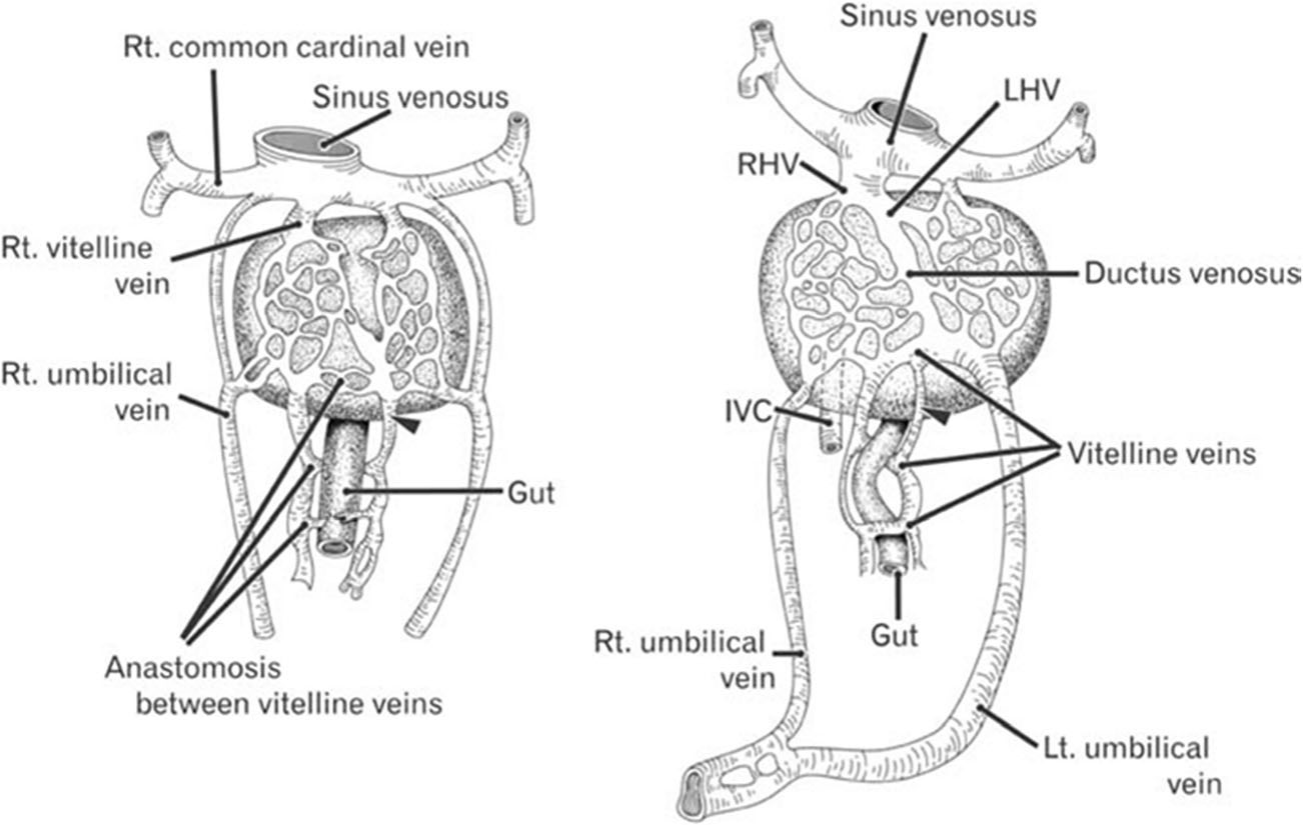
Diagram showing development of the portal and hepatic veins. The two vitelline veins communicate inside the liver and around the duodenum to form intrahepatic portal and hepatic veins: the left vitelline vein disappears, and the cranial part of the right vitelline vein and the segment that lies inferior to the liver give rise to the terminal branch of the IVC and portal and superior mesenteric veins. Incomplete involution and persistent communication of the vitelline venous system during the development of newly formed hepatic sinusoids results in various types of portosystemic shunts [11]

The development of the portal system is complex and occurs between the 4th and 10th week of embryonic life. Initially, the two vitelline veins emerge from the anterior surface of the yolk sac and drain to the sinus venosus. By the end of the 4th week, they create at least three cross-communicating channels (subhepatic-cranioventral duodenal, intermediate-dorsal duodenal, and caudal-ventral duodenal) around the developing duodenum in order to form the vitelline venous network. In the septum transversum, cords surround the intrahepatic component of the newly developing vitelline system in order to give rise to hepatic sinusoids, which selectively involute to form the final configuration of intrahepatic branches of portal and hepatic veins at 8th week [7–9]. Between the 10th and 12th week, the left vitelline vein disappears, and the cranial part of the right vitelline vein and the segment that lies inferior to the liver give rise to the terminal branch of the IVC and portal and superior mesenteric veins, respectively [7–10] (Fig. 1).

Incomplete involution of the vitelline venous system in response to the development of hepatic sinusoids is probably the main reason for shunt formation and depends on the anatomical site (right or left) and level (proximal or distal) at which the vitelline veins fail to differentiate. Intrahepatic shunt type 1 and extrahepatic type 2 side to side variants arise from persistence of the right vitelline vein, whereas other extrahepatic shunts draining into the IVC above the level of the hepatic vein confluence, or to the right atrium appear to be due to persistence of the left vitelline vein. Other authors have suggested that failure of remodeling of the anastomotic channels between vitelline and subcardinal veins during the development of the IVC in the early embryonic stages may play a role in creating type 2 extrahepatic shunts [8, 12, 13].

Extrahepatic shunt type 1 (Abernethy malformation) with the “absence” of the intrahepatic portal veins is thought to be the result of excessive involution of the periduodenal vitelline plexus. Similarly, persistent communication of the vitelline venules within the newly formed hepatic sinusoids results in type 2–4 intrahepatic shunts [8].

The ductus venosus connects the umbilical vein and the inferior vena cava during embryonic life. It arises from the posterior aspect of the left portal vein PV, opposite the opening of the umbilical vein and drains into the left hepatic vein near its entry into the IVC (or directly to the IVC) [14]. Spontaneous closure begins immediately after birth and is completed during the first week of life. Delayed closure may occur due to an alteration in hemodynamics from congenital heart defects, and a patent ductus venosus acts as an intrahepatic shunt and may result in hypoplasia of the portal vein [15, 16].

## Other abnormalities associated with CPSS

CPSS are associated with multiple congenital abnormalities, with the most common involving the cardiovascular system, and include ventricular and atrial septal defects (VSD, ASD), patent foramen ovale, coarctation of the aorta, tetralogy of Fallot, and patent ductus arteriosus (PDA). [16]. They potentially affect liver hemodynamics and contribute to the creation and maintenance of these portosystemic shunts (e.g., cardiac defects and patent ductus venosus) [16].

Another common abnormality is the polysplenia syndrome with azygos or hemiazygos continuation of the inferior vena cava, which is found in 8% of extrahepatic shunt cases. Screening for CPSS should be performed in all patients with polysplenia [3, 17]. Other vascular abnormalities associated with CPSS include splenic artery aneurysms, coronary artery fistulas, primitive hypoglossal artery, and cutaneous hemangiomas [18].

Other abnormalities and syndromes, which have been associated with CPSS, are summarized in Table 1. The majority have been associated with extrahepatic shunts, whereas anomalies with intrahepatic shunts are less frequent and limited to cardiac, renal, and biliary anomalies, vascular aneurysms, and a small number of rare syndromes (e.g., Trisomia 21, Leopard, and Rendu-Osler-Weber) [3, 7, 19, 21].

**Table 1:** Congenital anomalies associated with CPSS [3, 7, 18–20]

Cardiovascular
– Atrial or ventricular septal defect
– Patent foramen ovale
– Dextrocardia or mesocardia
– Congenital stenosis of aortic or pulmonary valves
– Tetralogy of Fallot
– Tricuspid atresia
– Mitral atresia
– Double inferior vena cava
– Left-sided inferior vena cava
– Azygos and hemiazygos continuation
– Skin hemangiomas
– Splenic artery aneurysms
– Coronary artery fistulas
– Primitive hypoglossal artery
Hepatobiliary
– Biliary atresia
– Annular pancreas
Urogenital
– Renal agenesis
– Cystic dysplasia of the kidneys
– Bilateral ureteropelvic obstruction of the kidneys
– Vesicoureteral reflux
– Crossed fused renal ectopia
– Hypospadias
Gastrointestinal
– Juvenile polyposis
– Duodenal atresia
Genetic syndromes
– Down, Bannayan-Riley-Ruvalcaba (macrocephaly and hemangiomas associated with hamartomatous polyposis syndromes), Turner, Holt-Oram, Grazioli and Goldenhar (skeletal malformations), Leopard, Rendu-Osler-Weber, Noonan
Miscellaneous
– Polysplenia, situs inversus

## Classification

Classification of CPSS can be challenging as significant variety and complexity, in terms of localization, configuration, size, vessels involved, number of pathways, and the presence of intrahepatic portal branches, exists. CPSS were historically classified by their anatomy; however, continued improvements in our understanding of the pathophysiology of the condition are helping to guide management. Extrahepatic shunts have also been classified according to criteria based on their clinical presentation and liver histopathology, which guide subsequent treatment [22].

For intrahepatic portosystemic shunts, the classification described by Park et al. appears to be the most descriptive and defines them as communications > 1 mm in diameter between the intrahepatic portal vein and the hepatic or perihepatic veins. Four types have been identified: a single vessel communication, which can be either between a main branch of the portal vein and IVC (type 1), peripheral location in one segment (type 2), or through an aneurysm (type 3), and multiple small communications distributed diffusely in both lobes (type 4) [23]. They appear to have a male predominance, with the first two varieties being the most common. A patent ductus venosus is invariably referred to as an intrahepatic shunt type 5, despite its course in the ligamentum venosum, because it originates from the left portal vein [11].

In the classification of extrahepatic portosystemic shunts, no difference has been reported in the incidence between male and female patients [20]. Based on evidence of portal flow to the liver, extrahepatic portosystemic shunts (EPSS) have been classified into type 1 “Abernethy malformations” with an end to side shunt and an apparent absence of any portal branches to the liver, and type 2 in which portal flow is partially diverted to systemic circulation with either a preserved or hypoplastic main portal trunk connecting to the IVC in a side to side fashion [24]. Type 1 can be further subdivided based on whether the superior mesenteric and splenic vein drain separately (type 1a) or via a common trunk (type 1b) to IVC or less commonly to another systemic vein (e.g., azygos, iliac, renal) [25].

A critical factor in the classification of CPSS is the presence of portal flow to the liver which can be determined by the evidence of patency of extrahepatic and intrahepatic portal branches. Assessment depends on cross-sectional imaging, the use of an occlusion test, and results from liver biopsy to understand the degree of hypoplasia of the intrahepatic portal venous system [26–28]. Complete extrahepatic or intrahepatic shunts, which with conventional imaging appear to show an absent portal flow to the liver, may reveal a previously hypoplastic intrahepatic portal system which gradually opens up after venogram and shunt occlusion. This has been proposed as an essential aspect of early assessment and management of all cases [26–28].

Other authors have proposed additional classification patterns in their effort to understand the pathophysiology of the condition and to help guide management. Kanazawa et al. proposed an alternative classification, based on the severity of the intrahepatic portal hypoplasia (mild, moderate, and severe) and the portal pressure at shunt occlusion, which would reflect the clinicopathological features and provide useful information about likely response to therapy (Table 2) [29]. Other classifications include those described by Lautz et al. based on the origin of the shunt in the portal circulation (Table 3), and Kobayashi et al. whose classification was based on the systemic site of drainage in relation to symptoms etiology (gastrointestinal bleeding, encephalopathy, liver tumors) (only extrahepatic Table 4) and that of Blanc et al. which focused on the shunt caval ending and the indications for surgical closure in either one or two stages based on the communication pattern (end to side vs side to side) (Table 5) [19, 30, 31]. A more detailed and accurate but possibly less practical anatomical description of both intra- and extrahepatic shunts was reported by Bernard et al. who reviewed 265 pediatric cases and classified them according to the site of shunt origin (portal vein, afferent and efferent branches), the ending systemic vein drainage, and the pattern and number of communications [3].

**Table 2:** CPSS classification according to the severity of the hypoplasia of intrahepatic portal system under shunt occlusion (Kanazawa et al. [29])

Type	Hypoplasia of intrahepatic portal system under shunt occlusion
A	Mild
B	Intermediate
C	Severe

**Table 3:** CPSS classification according to shunt origin (Lautz et al. [19])

Type of shunt	Origin and communication pattern
I	End to side portocaval shunt with no portal flow to liver
IIa	H type shunt arising from left or right portal vein (including patent ductus venosus)
IIb	H type shunt arising from main portal vein
IIc	H type shunt arising from mesenteric, splenic, or gastric veins

**Table 4:** CPSS classification according to shunt ending (Kobayashi et al. [30])

Type	Shunt ending and correlation with complications
A	IVC (liver nodules and encephalopathy)
B	Renal veins (encephalopathy)
C	Iliac veins (gastrointestinal bleeding)

**Table 5:** CPSS classification according to shunt caval ending (Blanc et al. [31])

Type	Caval ending
Portocaval end to side	IVC portion between hepatic vein and above renal veins
Portocaval side to side	IVC portion between hepatic vein and above renal veins
Portocaval H-shaped	IVC portion between hepatic vein and above renal veins
Persistent ductus venosus	Left hepatic vein
Portohepatic	Hepatic veins
Extrahepatic	IVC portion below renal veins (direct ending or via another systemic vein, e.g., left renal, iliac, etc)

## Clinical picture

Patients with CPSS present with a wide spectrum of symptoms and complications that may occur during life, although asymptomatic cases, discovered incidentally on imaging, are not uncommon. Hepatic encephalopathy, hepatopulmonary syndrome, and pulmonary hypertension are the most prominent manifestations caused by long-term portosystemic shunting and are more often observed in children [20, 32].

Even before birth, alterations in fetal venous circulation from shunting may result in decreased liver perfusion and signs of intrauterine growth restriction in the absence of hypoxia or other obvious maternal infections and/or chromosomal abnormalities [33]. Neonatal cholestasis and galactosemia may occur and should be differentiated from other congenital defects such as biliary atresia and metabolic disorders which may also coexist [34, 35].

Children with CPSS may present with unexplained neurocognitive dysfunction and other behavioral issues due to low-grade hepatic encephalopathy and this accounts for between 17 and 30% of cases [8]. Other manifestations include learning disabilities, extreme fatigability, seizures, and failure to thrive and have been associated with elevated arterial ammonia levels in the majority of cases. The likelihood of encephalopathy increases with age and is related to the shunt flow [8, 14].

Refractory hypoxia and hepatopulmonary syndrome can be found in about 10% of cases. Potent vasoactive mediators when bypassing the liver cause intrapulmonary vascular dilatation and impaired oxygen exchange. Patients usually present with cyanosis, digital clubbing, and dyspnea on exertion or at rest [36]. Associated portopulmonary hypertension can affect 13–66% of children with hepatopulmonary syndrome and CPSS and usually appears later in the course of the disease [7, 37, 38]. The histological picture is consistent with obliteration of pulmonary arteries with microthrombi and intimal fibrosis. The degree of hypertension does not seem to correlate with the shunt size and may possibly be secondary to a coexisting cardiac anomaly. Portopulmonary hypertension carries a poor prognosis with a reported mortality of 50% due to late identification and failure to reverse even after shunt closure [7, 37, 38].

Regenerating liver nodules (e.g., adenoma, focal nodular hyperplasia, hemangioma) have been reported as a result of the alteration in local hemodynamics, with the compensatory increase in arterial flow, and associated elevated circulating levels of hepatic growth factors (e.g., insulin, glucagon, hepatocyte growth factor) are seen in 25–50% of CPSS cases [39, 40]. They can be single or multiple, can occur at any age, and are most commonly found in adult patients with EPSS. Malignant tumors (hepatocellular carcinoma (HCC), hepatoblastoma, sarcoma) have been reported to occur in these patients in the absence of liver dysfunction and cirrhosis (4% of all cases) [41]. They appear exclusively with extrahepatic shunts and may occur as de novo primary tumors, but transformation of preexisting benign lesions seems to be more common. Hepatoblastomas are rare tumors and are associated with other genetic disorders as well. Cases in which they occur after transformation of preexisting focal nodular hyperplasia have been reported [41]. The risk of primary HCC in patients with extrahepatic shunts seems to be similar to that of liver cirrhosis. In most cases, the alpha-fetoprotein is elevated and the shunt appears to work as an independent risk factor in the absence of chronic liver disease [42, 43]. In addition, benign tumors such as adenoma or focal nodular hyperplasia show a different pathogenesis and natural history than conventional tumors and seem to carry a higher risk of malignant transformation. This is supported by the evidence of specific mutations (beta-catenin mutations resulting in activation of various transcription factors) discovered in hepatocytes possibly in association with the altered hemodynamics. For these reasons, indications for treatment differ and close surveillance of those nodules is recommended [44].

Gastrointestinal bleeding as a presenting symptom has been reported in 8.1% of cases with EPSS. In the majority of these cases, the ending systemic veins of the shunt were the iliac veins, resulting in colonic and rectal varices [45]. Encephalopathy and liver tumors were uncommon in this group of patients perhaps suggesting that the shunt was only partial and provided a degree of protection.

Children that were undiagnosed or put under long-term surveillance due to mild symptomatology and remained without any treatments may develop clinical symptoms at a later age with the likelihood to increase over 50 years. Adults usually show mild to moderate neurological impairment with features similar to patients with chronic liver disease. Other symptoms such as abdominal pain and hypoglycemia may be seen, too. Unusual findings such as parkinsonism, autism, and spastic paraparesis have also been described in association with hyperammonemia [46–48]. Asymptomatic patients with a low shunt function may develop symptoms of encephalopathy when precipitating factors such as gastrointestinal bleeding or constipation elevate blood ammonia levels [46–48].

Complications that have been reported with CPSS include hyperandrogenism caused by hyperinsulinemia due to insulin resistance from the altered liver hemodynamics (in a similar manner to liver cirrhosis), pancreatitis (associated anatomical narrowing of the pancreatobiliary junction), vaginal bleeding, and lower urinary tract symptoms (lithiasis, hematuria). A rare complication that has been described is membranoproliferative glomerulonephritis (MPGN), a well-known manifestation that usually can be seen in cirrhotic patients postdecompression of the portal system (e.g., surgical portocaval shunt) and subsequent reduced hepatic clearance of immune complexes [37, 49, 50].

## Diagnosis

A comprehensive workup comprising radiological, biochemical, and dynamic invasive tests are invariably required to establish the diagnosis and delineate the shunt anatomy. The selection of each test depends on the age of the children, the anatomical complexity of the shunt, the clinical signs and complications, and the potential for treatment at the same time. The initial tests should include Doppler ultrasound and arterial ammonia levels. Enhanced color Doppler ultrasound will also allow for estimation of the shunt ratio by dividing the blood flow volume at the shunt orifice by the total portal flow [51]. In the past, rectal scintigraphy was used to calculate the ratio uptake of rectally delivered isotope (iodine 123 iodoamphetamine) between the lungs and the liver as an indirect measurement of the amount of blood that passes through the shunt. Shunt ratios of greater than 5% were considered abnormal [13, 15]. This method has gradually been replaced by the Doppler ultrasound although one study demonstrated (Yuki Cho et al.) superiority in the estimation of shunt severity using rectal scintigraphy [12, 14, 52].

The level of serum ammonia varies, and although it is proportional to the degree of shunt flow and normalizes after shunt occlusion, studies have shown that it does not always correlate with the degree of encephalopathy [7, 8]. Moreover, it has been reported to be normal in some patients even in the presence of overt neurological symptoms. Repeated measurement of ammonia levels is required.

Additional tests may support further the diagnosis and help in assessing the severity of symptoms (e.g., encephalopathy). Newborn children may be diagnosed with a CPSS on routine screening for galactosemia without enzyme deficiency [34]. An oral glutamine challenge test may precipitate encephalopathy and define the problem more clearly. Magnetic resonance imaging of the brain may reveal white matter atrophy and abnormal signals on the T1-weighted images of the basal ganglia, characteristically at the globus pallidus, which correspond to manganese deposition. This finding has been reported in the absence of clinical encephalopathy in a patient with CPSS. Electroencephalography and other neuropsychological tests are invaluable, as low-grade encephalopathy is often misdiagnosed as behavioral problems [8, 21, 53].

Abdominal imaging (computed tomography or magnetic resonance imaging) is used initially to delineate shunt anatomy and characterize potential focal liver lesions. This may indicate the absence of intrahepatic portal network; therefore, in such cases, invasive tests (e.g., mesenteric portovenography) are required to evaluate the intrahepatic portal system plasticity via an occlusion test. Definitive occlusion of the shunt at the same time, depending on portal hemodynamics, is an option for favorable cases. If this is not feasible, data from the occlusion test can be utilized for planning future intervention and final treatment modality [7, 54].

Liver biopsy may show atrophy of the liver, due to portal flow deprivation and lack of hepatotrophic factors; however, findings of advanced fibrosis or cirrhosis are rarely if ever seen. Complete or near complete absence of the portal venules and hypertrophy of hepatic artery branches and bile duct proliferation, with or without nodular regenerative hyperplasia, are common finding especially with type 1 extrahepatic shunts. These findings help in predicting the potential for expansion of portal venules after shunt occlusion [54]. According to Kanazawa et al., the severity of intrahepatic hypoplasia of the portal network correlates with the size and not the number of portal triads as it was found that the number was similar in the mild, moderate, and severe types of hypoplasia. Small-sized portal triads were seen extensively in the severe type as opposed to the moderate (occasionally) and mild types (rarely) in which portal venules were constricted and distorted in a crescent shape. In this study, 13 patients with mild or moderate hypoplasia and 2 with severe hypoplasia tolerated definitive radiological occlusion of an extrahepatic shunt in a single procedure [29]. Liver biopsy in the setting of an indeterminate or increasing size liver nodule may also be necessary. Serum alpha-fetoprotein levels are mandatory in helping to evaluate focal liver lesions or screen at-risk patients in combination with ultrasonography. In addition, other tests may be useful to assess potential underlying liver disease especially in adult patients with spontaneous shunts (elastography, hepatitis screen, etc) [41, 54–57].

Further tests to identify and assess potential complications in other organs or to investigate associated congenital anomalies (e.g., cardiac echo, GI endoscopy, lung studies) are routinely used.

## Treatment

CPSS are rare abnormalities and large series which have adopted a standard therapeutic approach are not currently available. The shunt type, location and degree of function, patient age, and the severity of symptoms and complications determine treatment strategy. Conservative management has been proposed in cases with mild symptoms or when spontaneous closure was anticipated. The basic principle of intervention is to disrupt the abnormal communication between portal and systematic circulation and restore portal flow to the liver. The view has emerged that all shunts that persist after the first year of life should be closed without waiting for complications to develop.

Observation and monitoring of arterial ammonia levels may be sufficient in asymptomatic adult patients with low flow shunts who can be followed up with serial Doppler ultrasound [58]. The likelihood of symptoms is proportional to the shunt size and flow (> 30%). Medical management is similar to that used for cirrhotic patients with hepatic encephalopathy including protein restriction, lactulose, and nonabsorbable antibiotics. Previously, it was recommended that monitoring of the shunt size and ammonia level was sufficient for mild symptomatology. However, this has changed and these shunts should also be closed after excluding the presence of significant pulmonary hypertension [58].

Intrahepatic shunts that are diagnosed prenatally or during early infancy do not necessarily require definitive treatment, as many will spontaneously close by the age of 1 year with resolution of symptoms, in contrast to extrahepatic shunts and patent ductus venosus where closure is unlikely [59]. Spontaneous closure of intrahepatic shunts is more often seen in girls, in the presence of multiple shunts and in children with neonatal cholestasis [59].

For children with EPSS or a persistent intrahepatic portosystemic shunt, the optimal timing of treatment has not been defined. Many authors have proposed that even in the absence of overt symptoms, early intervention prevents pulmonary complications and neurodevelopmental delay and intellectual and psychosocial function may be preserved [22]. The presence of clinical encephalopathy, hepatopulmonary syndrome, portopulmonary hypertension, regenerative liver nodules, and evidence of increasing shunt size are clear indications for intervention if the patient is fit enough to tolerate any planned procedure. Cases with refractory symptoms, previous failed medical treatment, or an increased shunt ratio > 60% are likely to benefit from shunt occlusion. The choice of radiological or surgical approach depends on local expertise, shunt anatomy and size, and the patient’s fitness [22, 29].

Interventional radiology is the least invasive method and results in rapid amelioration of symptoms, correction of high ammonia levels, and regression of liver lesions [32, 60, 61]. Catheter insertion via a transhepatic route offers better exposure for a intrahepatic shunt associated with the contralateral portal vein to the initial punctured entry side, when a transcaval route, using either common femoral or internal jugular vein access, is preferred for large intrahepatic shunts close to the inferior vena cava or any type of extrahepatic shunts. A small number of cases using a transileocolic approach via a minilaparotomy have also been described [9].

Embolization of the shunt using various materials including coils and microcoils, detachable balloons, and *N*-butyl cyanoacrylate lipiodol mixtures or recently introduced multilayer devices (vascular plugs) has been reported depending on shunt size [32, 62, 63]. For small caliber shunts, coils are effective and the risk of migration is low. For larger, high-velocity flow shunts, a vascular plug (e.g., Amplatzer) with a diameter 30 to 50% larger than the fistula size has been used and can be positioned accurately with less metallic artifact on follow-up imaging. This allows better visualization of any persistent venovenous shunting. The combination of more than one material has also been reported as being effective in recurrent or complex shunts when a vascular plug positioned first can be used as an anchor for coils that target small vessels with residual flow [63].

During the procedure, an occlusion test with a temporary balloon placement is recommended to record portal vein pressure elevation. If previous liver biopsy has demonstrated severe hypoplasia of intrahepatic portal system, then portal pressure is expected to be high [38, 64–66]. If the portal pressure exceeds 30 mmHg, then permanent occlusion at this stage should be avoided, as it is likely to cause significant changes in liver hemodynamics, derangement of liver function, and potential worsening of preexisting symptoms and gastrointestinal bleeding. A two-stage approach is advocated for these cases with the use of a size reduction stent followed by definitive occlusion (radiological or surgical ligation) a few months later and will allow the liver to compensate and the portal pressure to reduce gradually without complication [38, 64–66].

Surgical ligation is an acceptable method and can be used in cases with large shunts with a risk of inadvertent migration of embolic agents during embolization or after failed embolization with shunt recurrence or persistence. Moreover, if the shunt is not long enough as in a side to side communication, then placement of coils or plugs may be difficult [15, 29, 67, 68]. Laparoscopic ligation has also been reported in more peripheral extrahepatic shunts (e.g., splenorenal) [69]. Surgical ligation has some relative limitations in terms of failure to identify intrahepatic or multiple shunts and the risk of acute portal hypertension/thrombosis and subsequent bowel congestion. Blanc et al. have proposed a classification for CPSS based on the largest series of surgical ligations in one or two stages. The authors emphasized that ligation should be as close as possible to the caval system as inadvertently proximal severe portal hypertension and thrombosis of blind portal segments may occur during occlusion. If portal pressure is high (cutoff point 25 mmHg), then temporary banding and completion of ligation in two stages is preferable. End to side shunts are no longer considered as contraindications for surgical ligation because of potential significant revascularization of the intrahepatic portal system [3, 31, 54, 69].

Liver resection or transplantation has been performed for the treatment of extrahepatic or large intrahepatic multifocal shunts not amenable to embolization or in cases of previous failed radiological intervention or where HCC or hepatoblastoma has developed. Shunts with tumors causing obstruction, have a multifocal distribution, are rapidly increasing in size/changing features, or malignancy is suspected require resection with definitive histological diagnosis and potential further treatment. Such tumors may develop at any age even without prior shunt symptomatology [70]. Embolization of HCC is associated with significant ischemic injury (due to the lack of portal vein inflow) and, while very effective, should be used more cautiously. Indication for combined ligation and resection of concomitant malignant liver tumor may also exist in an extrahepatic shunt where resection alone would leave the shunt untreated [41]. Liver transplantation remains the only option for type 1 extrahepatic shunts which maintain increased portomesenteric pressure and fail to establish a sufficient intrahepatic portal network, after temporary shunt occlusion. In some type 1 extrahepatic shunts where radiological occlusion initially seems favorable, “abnormal” cavernous transformation of portal system indicates a nonphysiological remodeling with equivocal results without resolution of symptoms in the longer term. Indications for liver transplant remain poorly defined except for HCC cases [71]. Sakamoto et al. have reviewed 34 patients treated by liver transplantation (both deceased and living donor) for extrahepatic shunts. Indications included hepatopulmonary syndrome, hyperammonemia with encephalopathy and liver tumors. Despite technical difficulties due to abnormal anatomy especially with the portal vein anastomosis, good outcomes were reported, with 31 patients alive (91.2%) at a median follow-up of 18 months [71, 72].

**Fig. 2:**
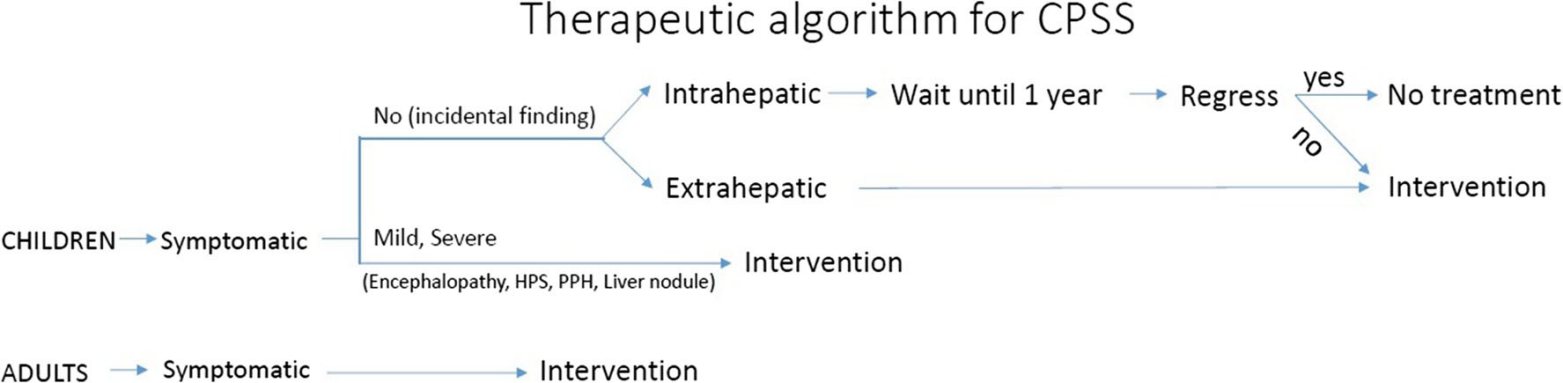
Therapeutic algorithm for CPSS

**Fig. 3:**
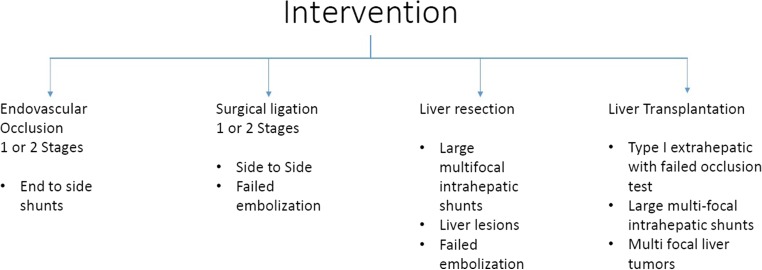
Intervention protocol for CPSS

Although overall management may differ among different centers and there is no universal treatment protocol, we are proposing a therapeutic algorithm for all the CPSS which is shown in Figs. 2 and 3. We suggest that all extrahepatic shunts should receive treatment regardless of their symptoms as early intervention has been shown to prevent complications and may preserve intellectual and psychosocial development. For intrahepatic shunts even in the presence of overt symptomatology, observation until 1 year of age should be offered as spontaneous regression may occur. In cases where a congenital shunt is left untreated or not diagnosed until adulthood, indications for treatment depend on the severity of symptoms. First-line treatment currently is endovascular occlusion of the shunt in 1 or 2 stages, reserving surgical options (ligation, resection, transplantation) for those shunts that are not amenable or fail to embolize, or are associated with liver tumors (Figs. 2 and 3).

## Conclusion

CPSS are rare embryologic abnormal communications between the portal and systemic venous circulations and are associated with other congenital anomalies. Liver tumors, pulmonary vasculature complications, and hepatic encephalopathy are common. Early recognition and correction with either radiological or surgical occlusion reverses symptoms and prevents long-term complications. Large series with a standard therapeutic approach are not available as yet. Continuing the focus on the pathophysiology and anatomy of these lesions will help guide future management.

## Abbreviations

CPSS: Congenital portosystemic venous shunt
EPSS: Extrahepatic portosystemic shunt
HCC: Hepatocellular carcinoma
IPSS: Intrahepatic portosystemic shunt
